# Immune globulin therapy and kidney disease: Overview and screening, monitoring, and management recommendations

**DOI:** 10.1093/ajhp/zxac139

**Published:** 2022-05-20

**Authors:** Roger H Kobayashi, Michael T Rigas

**Affiliations:** Pediatric Immunology and Allergy, University of California, Los Angeles, School of Medicine, Los Angeles, CA, USA; KabaFusion, Cerritos, CA, USA

**Keywords:** chronic kidney disease, high-dose immune globulin, immune globulin, intravenous immune globulin, renal impairment, subcutaneous immune globulin

## Abstract

**Purpose:**

This report calls attention to the potential risks of diminished kidney function when administering immune globulin (IG). The goal is to increase awareness of chronic kidney disease (CKD) and kidney function impairment in patients receiving IG and provide recommendations for screening, monitoring, and management to promote risk prevention and mitigation.

**Summary:**

Human IG preparations for intravenous (IVIG) or subcutaneous (SCIG) administration are the mainstay of treatment in patients with primary immunodeficiency diseases. Increasingly, IVIG at high doses (1,000 to 2,400 mg/kg) is also used as a treatment for a variety of autoimmune and inflammatory conditions. Although some autoinflammatory disorders respond to a single course of IVIG therapy, the majority of patients require long-term, regular infusions, thereby increasing the overall risks. Often, both patients and physicians treating adults with IG are unaware of underlying CKD or kidney function impairment. This lack of awareness constitutes a major risk factor for potential worsening, particularly when using high doses of IVIG. Therefore, screening of all patients for CKD and kidney function impairment before the use of IG is essential. Identification of the cause of kidney impairment is strongly encouraged, as IG therapy may need to be modified.

**Conclusion:**

As detailed here, there are potential risks to patients with impaired kidney function with administration of IG, particularly at high doses. Product selection, volume, route of administration, and rate of infusion may impact those with compromised kidney function. Therefore, screening of all patients for CKD and kidney function impairment before the use of IVIG and SCIG, as well as ongoing monitoring and management, is critical. As with all potential adverse drug reactions, the best approach is to prevent them.

**KEY POINTS**

- Clinicians should evaluate all patients receiving immunoglobulin (IG) for underlying chronic kidney disease (CKD) and kidney function impairment on a regular basis; this is of critical importance in patients receiving high-dose intravenous IG.
- If CKD or kidney function impairment is identified, patients receiving IG should be closely monitored and managed to prevent further damage to kidney function.
- Identification of the cause of CKD or kidney function impairment is strongly encouraged, if feasible, as IG therapy may need to be modified.

Human immune globulin (IG) preparations for intravenous (IVIG) or subcutaneous (SCIG) administration are the mainstay of treatment in patients with primary immunodeficiency diseases (PIDDs) as a replacement therapy.^1^ Administration of IG is an essential and life-saving therapy as these patients are unable to mount an effective immune response against infections. All IG products currently approved by the Food and Drug Administration (FDA) include an indication for IG replacement in PIDDs.^2^ Treatment with IG has also been FDA approved for a variety of autoimmune and inflammatory conditions, including immune thrombocytopenic purpura (ITP), chronic inflammatory demyelinating polyneuropathy (CIDP), dermatomyositis (DM), and multifocal motor neuropathy (MMN).^3^ Because of its immunomodulatory and anti-inflammatory effects, IG is also frequently administered off-label for a number of rare disorders and provides significant benefit in both acute and chronic conditions as a steroid-sparing agent and maintenance therapy.^3^ In the US, more than 75% of IVIG is administered to patients with autoimmune or inflammatory conditions.^4^ Depending on the specific autoimmune or inflammatory disorder being treated, typically high-dosage regimens and frequencies are used. Although some disorders can respond to a single course of IG therapy, the majority of patients require long-term, regular (weekly or monthly) infusions, thereby increasing overall risks.

In many instances, IG has been life-saving; however, administration of IG can also lead to mild, as well as severe, adverse reactions, including aseptic meningitis, thromboembolic events, hemolytic reactions, and anaphylaxis.^3^ The use of IVIG has also rarely been associated with serious systemic adverse reactions, including kidney failure.^3^

Approved IG products are manufactured via a fractionation process of pooled plasma from thousands of screened donors and undergo complex pathogen inactivation and purification steps to optimize product safety while ensuring preservation of functional activities.^5^ This report is focused on the potential risks with use of IVIG in patients with impaired kidney function. The goal is to increase awareness and provide recommendations for assessment, monitoring, and early treatment to promote risk prevention and mitigation.

## Overview of kidney disease

### Classification, prevalence, and risk factors.

The Kidney Disease: Improving Global Outcomes (KDIGO) group has published guidelines that define chronic kidney disease (CKD) as kidney damage and/or a decreased glomerular filtration rate (GFR) of less than 60 mL/min/1.73 m^2^ for at least 3 months (if the duration of decreased GFR is less than 3 months or unclear, patients may have CKD or kidney impairment and tests should be repeated accordingly).^6,7^ The different stages of classification of CKD form a continuum (Box 1). By itself, GFR may not be sufficient to identify stage 1 and 2 CKD, as it may be normal or borderline normal. It is recommended that GFR and albuminuria levels be used together, rather than separately, to improve prognostic accuracy in the assessment of CKD. More specifically, the guidelines recommend the inclusion of estimated GFR and albuminuria levels when evaluating risks for overall mortality, cardiovascular disease, end-stage kidney failure, and acute kidney injury (AKI) and the progression of CKD.^6,7^

**Box 1. Stages of Chronic Kidney Disease**

**Stage 1:** kidney damage (eg, albuminuria) with normal or increased GFR (>90 mL/min/1.73 m^2^)

**Stage 2:** kidney damage (eg, albuminuria) with mild reduction in GFR (60-89 mL/min/1.73 m^2^)

**Stage 3a:** mild to moderate reduction in GFR (45-59 mL/min/1.73 m^2^)

**Stage 3b:** moderate reduction in GFR (30-44 mL/min/1.73 m^2^)

**Stage 4:** severe reduction in GFR (15-29 mL/min/1.73 m^2^)

**Stage 5:** kidney failure (GFR <15 mL/min/1.73 m^2^ or dialysis)

Abbreviation: GFR, glomerular filtration rate. Adapted with permission from reference 6.

CKD is an increasing public health issue in global populations.^8^ Approximately 15% of adults in the US (or 37 million people) are estimated to have CKD (including stages 1-4; stage 5 is not included in this estimate).^9^ Worldwide, prevalence is estimated at 8% to 16% in adults.^8^ Patients with CKD in stages 1 to 3 are generally asymptomatic. Most (9 in 10) adults are unaware of their CKD status, and about 2 in 5 people with severe CKD who are not on dialysis are unaware of underlying impaired kidney function.^9^ Diabetes mellitus and hypertension are the major causes of CKD in adults in developed countries.^8,10^ People may not feel ill or notice any symptoms until CKD is advanced.^9^ Therefore, recognition of the increased risk of CKD is vital. Risk factors include advanced age, family history of kidney disease, history of AKI, reduced nephron mass (history of nephrectomy or low birth weight), kidney damage with normal or increased GFR (>90 mL/min/1.73 m^2^), and genetic traits.^10^ In addition, exposure to diseases, conditions, and/or medications that cause CKD (eg, autoimmune diseases, infections, iodinated radiographic contrast, and aristolochic acid) is also a risk factor.^10^

CKD is viewed as a significant contributor to the rising worldwide noncommunicable disease burden.^8,9^ Key risk factors for CKD are also increasing in prevalence, including obesity, which predisposes individuals to diabetes mellitus and hypertension (see “IG and impaired kidney function: screening, monitoring, and management recommendations”). The risks for all noncommunicable diseases increase with elevated body mass index.^11^ The prevalence of obesity worldwide has nearly tripled since 1975, and more than 1.9 billion adults are considered overweight (approximately 39% of men and 40% of women).^11^ Once considered a problem of high-income countries, overweight and obesity are now on the rise in low- and middle-income countries, particularly in urban settings. As a result, CKD prevalence is expected to increase in parallel.

As emphasized in the previous section, the majority of adults are unaware that they have CKD.^8^ This lack of awareness on the part of patients and their physicians is a major risk factor for worsening of the disease. Therefore, screening of all patients for CKD before use of IG, as well as ongoing monitoring and management in patients with CKD, is essential. In addition, identifying the cause of the CKD is strongly encouraged; IG treatment may need to be modified according to the etiology of the impaired kidney function, which can influence the prognosis and relative importance of risk factors.^7^

## IG: indications and risk factors for kidney complications

### Indications for use.

Individuals with PIDDs are treated with IG to replace low or missing levels of antibodies and reduce infection rates.^12-20^ Dosing of IVIG and SCIG for replacement therapy in patients with PIDDs ranges from 300 to 800 mg/kg body weight.^3^ Utilization of IG has also been approved for several systemic autoimmune disorders owing to its immunomodulatory and anti-inflammatory effects, albeit at much higher doses (1,000-2,400 mg/kg) than those used for replacement therapy in PIDDs.^3,21-25^ In September 2008, FDA approved the first IVIG therapy for CIDP.^26,27^ Utilization of IVIG in CIDP was based on positive results in acute Guillain-Barré syndrome, as these disorders appear to be variants of the same immune-mediated inflammatory disorder of the peripheral nervous system.^28-30^ In 2012, IVIG was also approved for MMN.^31,32^ In addition to these approved, on-label uses, high-dose IVIG is also widely used off-label for a number of autoimmune and inflammatory disorders.^3^

Administration of IG via subcutaneous infusion, or SCIG, has also been approved in the US for PIDDs and select neurological indications, including CIDP.^33,34^ For patients who have experienced adverse reactions to IVIG, including kidney complications, SCIG may represent an acceptable alternative. Like IVIG, SCIG is also used off-label to treat autoimmune diseases.^35^ One of the main challenges with SCIG in autoimmune diseases is administering larger doses (as compared to replacement doses) and the limited volume of IG that can be infused at one time.^33,34,36^ However, because infusions can be given in the convenience of a home setting, patients adapt to more frequent infusions (ie, weekly or biweekly) with the added benefit of fewer systemic reactions and a steady, less fluctuating serum immunoglobulin G (IgG) level. The decision to utilize SCIG or IVIG is dictated by each patient’s disease characteristics, treatment goals, lifestyle, and insurance mandates.^34^ Because this report is focused on kidney complications associated with IG, local complications associated with SCIG are not covered.

### Key risk factors for kidney complications

Currently, all IG products are manufactured via a fractionation process in which plasma is pooled from thousands of screened donors and then undergoes highly complex, rigorous manufacturing processes ensuring pathogen inactivation, preservation of functional activities, and product safety and efficacy.^5^ Various excipients are added to stabilize IG products and prevent aggregation and dimer formation, including human albumin, glycine, polyethylene glycol, and sugars such as sucrose, maltose, and glucose.^3,37^ These excipients, also known as stabilizers or additives, have clinical implications.^38,39^ A full comparative analysis of all IG products is beyond the scope of this article; however, a comprehensive comparative chart of IG products is available from Managed Healthcare Associates, Inc., at https://bit.ly/32xBrEo (link provided with permission).

Onset of kidney impairment after IG administration is rare but is one of the most significant adverse reactions related to IG.^3,35^ The relationship between IVIG stabilized with sucrose and kidney impairment, including AKI, kidney failure, osmotic nephrosis, and kidney insufficiency, is now well known.^39-42^ Sucrose causes kidney injury as it enters tubular epithelial cells via pinocytosis. Epithelial cell swelling and cytoplasmic vacuolization occur as a result of incorporation of sucrose into lysosomes, which in turn causes narrowing of the tubular lumina. Thus, AKI induced by sucrose-containing IVIG may be related to the toxic action of sucrose, which causes degradation of the proximal tubular epithelium and kidney injury.^39^ The incidence of IVIG-related kidney impairment cannot be precisely determined, but the numbers of reported cases suggest a significantly lower incidence for products containing stabilizers other than sucrose.^39^

From 1981 (when IVIG was introduced) to November 1998, FDA received 114 reports worldwide of impaired kidney function and AKI associated with IVIG. The majority of patients recovered kidney function, but 17 deaths were reported following kidney failure.^43^ Almost 88% of these reports were associated with the use of sucrose-stabilized products. The French Regional Pharmacovigilance Center reported 49 cases of AKI that occurred between 1992 and 1998, all of which were associated with sucrose-stabilized preparations.^44^ This finding was initially reported in 1987 as acute cryoglobulinemic renal failure.^45^ Incidence of kidney impairment declined after FDA required all IVIG manufacturers to revise their prescribing information to include a black box warning.^43^ These data also triggered modifications of the stabilizers used in IVIG products and the development of sucrose-free products.

Unfortunately, some publications regarding IVIG stabilizers group sugars together and imply that their properties and clinical effects are all similar to those of sucrose, which is not accurate.^38,39^ For example, the clinical effects of sugar-stabilized IVIG preparations were assessed by Chapman et al.^46^ While being treated with a sucrose-stabilized IVIG preparation, a 44-year-old male developed AKI requiring hemodialysis. After his kidney function normalized, the patient was switched to a d-sorbitol–stabilized IG without any resulting kidney complications. d-Sorbitol is primarily metabolized within the liver and, to a lesser degree, in the kidney and other tissues. Intracellular accumulation of sorbitol is well regulated within the kidney by the complementary actions of aldose reductase and sorbitol dehydrogenase, which may account for the relatively low incidence of AKI with sorbitol-stabilized IVIGs.^47-49^

Maltose is a water-soluble disaccharide. Unlike sucrose, maltose is metabolized by kidney cells.^38,39,50^ IVIG products stabilized with maltose are well tolerated because maltase, the enzyme that hydrolyzes maltose to glucose, is present in the brush border of proximal convoluted renal tubules.^49^ Animal experiments have demonstrated that, unlike sucrose, for which more than 60% may be excreted unchanged in the urine, maltose is mostly metabolized, with less than 5% excreted unchanged.^51^ Conversion of maltose to glucose occurs intracellularly in the kidney; thus, maltose does not increase glucose levels in the blood. Although IVIG preparations stabilized with maltose have been associated with falsely high blood glucose meter readings in older systems based on glucose dehydrogenase pyrroloquinoline quinone or glucose-dye-oxidoreductase methods,^52^ this issue has largely been resolved with the use of modern glucose-specific blood monitoring methods.

Glucose is a monosaccharide that possesses a free ketone or aldehyde group, allowing it to donate electrons to other molecules and, thereby, act as a reducing agent.^38^ Excipient-grade d-glucose (dextrose) is frequently used for its various chemical properties (osmotic, diluent, and sweetening) and its ability to improve the stability of oxidation-sensitive active materials.^38^ Intravascular glucose is rapidly distributed to extracellular fluid and is expediently catabolized or converted to glycogen.^49^ Although glucose, when used as an excipient in IG preparations, does not appreciably increase glucose levels in blood, excessive glucose accumulation can have negative effects on proximal kidney tubules in humans.^49^ Because IV glucose infusion is known to produce a rapid increase in blood glucose and insulin levels in normal individuals, patients with diabetes may be at risk of kidney damage following administration of IVIG products stabilized with glucose.^53^

When kidney complications do occur, kidney insufficiency typically develops within 1 week (1-10 days) of the initiation of IVIG treatment.^54^ Common manifestations include hematuria and mild to moderate proteinuria.^55^ The serum creatinine level usually peaks around day 5 (range, days 3-8). Oliguric kidney failure is more common than nonoliguric kidney failure.^55^ In addition to risk factors directly related to product stabilizers, specific characteristics may predispose some patients to kidney complications following the use of IVIG (see “IG and impaired kidney function: screening, monitoring, and management recommendations”).

In most patients, AKI is reversible within 4 weeks (mean, 10 days) of discontinuation of the IVIG.^40,41,55,56^ However, in approximately 40% of patients, at least 1 day of hemodialysis is required.^40,41^ Most patients recover within 2 weeks of hemodialysis.^57^ Despite the eventual positive outcomes observed in most patients with IVIG-related kidney impairment, AKI may progress to CKD or kidney failure.^55^ Death may occur in 8% to 15% of these patients, but most have additional underlying conditions that make it difficult to determine precise causality.^40,43,58^

Many components other than stabilizers may influence tolerability in patients with kidney impairment, especially in those with late-stage CKD. For example, products vary in their osmolality, pH, and salt content. Osmolality ranges between 192 and 1,250 mOsm/L depending on the product, and the osmolality of the product must be considered when treating patients with impaired kidney function who may not tolerate a large osmotic load.^59^ The sodium chloride content ranges between 0% and 2%, and low-salt formulations are available for patients who are sensitive to fluid and/or salt. Products with a higher IVIG concentration may be advisable in patients with heart failure who may not tolerate excess fluid volume.^59^ Interestingly, high-dose IVIG has been successfully utilized for the suppression of panel reactive antibodies in the management of highly sensitized patients with late-stage CKD receiving dialysis while awaiting kidney transplantation.^59,60^

As with all potential adverse drug reactions (ADRs), the best approach is avoidance. To facilitate increased awareness among clinicians who administer IG, we have developed recommendations for screening, monitoring, and management for CKD to promote risk prevention.

## IG and impaired kidney function: screening, monitoring, and management recommendations

### Screening

**Figure 1.**
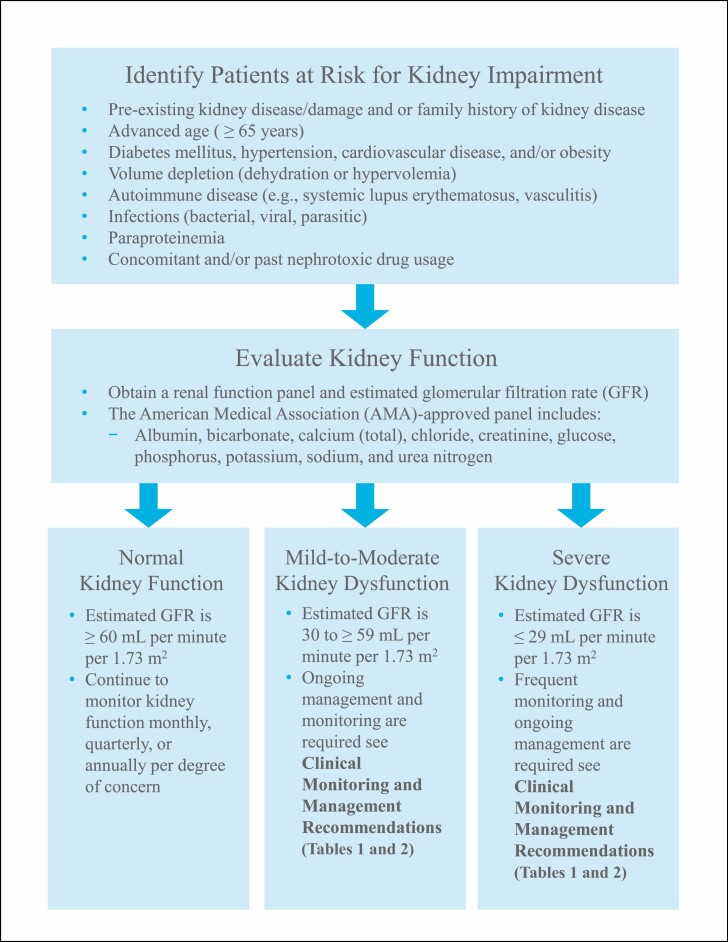
Screening for risk factors for kidney impairment.

Several risk factors for AKI and CKD after IG administration have been identified, including preexisting CKD or kidney damage or a family history of kidney disease, advanced age (≥65 years), diabetes mellitus, hypertension, obesity, cardiovascular disease, volume depletion (dehydration or hypervolemia), autoimmune disease, infections, paraproteinemia, and concomitant and/or past nephrotoxic drug usage (Figure 1).^39,40,43,55,58^ Therefore, all patients receiving IG should be screened for CKD risk factors and serum creatinine levels should be obtained before initial IVIG or SCIG administration (Figure 1 and eAppendix).

To date, there have been no reports of SCIG-induced kidney toxicity.^35^ The volume infused with each treatment is much lower with SCIG, thereby mitigating kidney toxicity. However, we propose screening for and management of potential kidney impairment in all patients receiving IG because of the relative lack of awareness of this widespread disorder.^61^

### Monitoring and management recommendations

Impaired kidney function can progress to late-stage CKD, kidney failure, and even death with the use of IVIG and SCIG. Early detection of CKD, especially in patients on high-dose IVIG therapy, is critical for initiating timely interventions and modifications, thus limiting the potential for further reduction in GFR. For patients with a history of impaired kidney function or risk factors for CKD, ongoing monitoring and management are recommended (Table 1, Table 2, and eAppendix).

**Table 1.** Immune Globulin Administration: Clinical Monitoring Recommendations for Patients With Impaired Kidney Function

Clinical monitoring	Recommendations
Routine laboratory evaluations	• Mild to moderate: renal function panel (Figure 1) and calculation of estimated GFR performed at least quarterly
	• Severe: renal function panel (Figure 1) and calculation of estimated GFR performed monthly or before every IVIG treatment
Changes in weight	• Carefully monitor fluid status, including decreases in urine output, edema, etc.
	• Obese patients should have their dose evaluated and monitored to avoid overestimating.
Medications	• Generally, it is suggested to reassess all concurrently prescribed drugs, particularly in older patients, on a regular basis.
	• Some concomitant medications should be used with caution in patients with impaired kidney function, eg, diuretics, inhibitors of the renin-angiotensin system, and nonsteroidal anti-inflammatory drugs.
Adverse reactions	• Carefully monitor for all adverse drug reactions during and up to 72 hours after infusion; resolve and mitigate them as soon as possible and minimize impact (Table 2).

Abbreviations: GFR, glomerular filtration rate; IVIG, intravenous immune globulin.

**Table 2.** Immune Globulin Administration: Clinical Management Recommendations for Patients With Impaired Kidney Function

Clinical management objective	Recommendations
Minimize further kidney injury	• If kidney function estimates show a reduction in creatinine clearance of more than 15%, weight gain of greater than 2.5 pounds, or increased edema, consider the following: ◦ Reducing infusion rate—consider a maximum infusion rate of 100 mL/h ◦ Spreading out dosing to every other day (or more), as needed (ie, decreasing daily volume) ◦ Assessing hydration and oral fluid intake before and after doses ◦ Evaluating and adjusting the current regimen of diuretics ◦ Carefully monitoring and assessing comorbidities • Delve deeper into the patient’s clinical changes and develop a more comprehensive care plan
Minimize the impact of ADRs	• If ADRs occur, adjust the care plan accordingly and consider the following: ◦ Introducing the interventions described above to minimize further kidney injury ◦ Introducing/modifying an IVIG premedication regimen ◦ Changing the concentration of IVIG product or IVIG brand if the ADR is significant ◦ Changing the administration location (with additional supervision), if appropriate ◦ Switching to SCIG therapy, if the patient is agreeable to this suggestion
Manage psychological and social issues	• Address the psychological impact and social burden of living with chronic kidney disease and consider the following: ◦ Therapeutic options for depression and mental health ◦ Provision of social services or home health support services ◦ Addressing anything else the patient is concerned about, eg, access to support, insurance, supplies, progress towards goals, etc
Minimize the impact of financial issues	• If changes to insurance or the ability to afford out-of-pocket costs occur, consult with the pharmacy intake team to develop a plan to ensure the therapeutic regimen is not interrupted

Abbreviations: ADR, adverse drug reaction; SCIG, subcutaneous immune globulin; IVIG, intravenous immune globulin.

One of the key elements of monitoring is routine laboratory evaluation of GFR at baseline with repetition at least quarterly for those with mild to moderate kidney disease and at least monthly for those with severe renal disease (Table 1). In addition, changes in weight, medications, and/or any ADRs require thorough evaluation. Specific management strategies to minimize kidney injury include reducing the infusion rate, increasing dosing intervals (decreasing daily volume), ensuring appropriate hydration, ongoing assessment of diuretics, careful monitoring and assessment of all comorbidities, and minimization of the impact of any ADRs that do occur (Table 2).

### Example case studies

To better understand the impact of kidney disease, specifically in relation to IVIG administration, illustrative case examples including case presentation and management are provided below.

#### Case study 1: IVIG for treatment of primary immunodeficiency in an older patient following AKI

A 65-year-old African American female was diagnosed with common variable immunodeficiency later in life following repeated upper respiratory tract infections. She had been stable on replacement IVIG for the last 10 years. She was recently hospitalized for a severe skin and soft tissue infection in her right upper arm and received vancomycin and piperacillin/tazobactam. An ADR to the antibiotic regimen resulted in AKI (GFR decreased from 76 to 32 mL/min/1.73 m^2^) that required a 2-week stay in the intensive care unit (ICU). She received daily hemodialysis in the ICU and was subsequently moved to the hospital floor and discharged 1 week later with an improved GFR of 63 mL/min/1.73 m^2^. However, because of her recent change in status, the physician recommended switching to SCIG to reduce the potential likelihood of any systemic adverse events, including any further decrease in kidney function. The patient and her family were trained on SCIG administration, and nursing staff plan to continue to assist the patient with the transition over the next few months. If her estimated GFR decreases further and/or she experiences any ADRs related to SCIG treatment, she will be reevaluated.

#### Case study 2: IVIG for CIDP in an obese patient with diabetes

A 35-year-old Caucasian male was diagnosed with CIDP in his late twenties and received high-dose IVIG (2,000 mg/kg), at a rate of 300 mL/h, on a monthly basis for approximately 15 years. Over the last 2 years, he gained a considerable amount of weight and was recently diagnosed with diabetes. He was prescribed metformin and is also trying to control his blood sugar with diet and exercise. In addition, the patient reported that he has very low energy and often feels depressed, which limits his motivation to exercise and maintain a healthy diet.

Following the screening recommendations, a kidney function panel was obtained. The patient’s estimated GFR was 42 mL/min/1.73 m^2^, indicating that he had a moderate reduction in GFR. According to the recommendations, his kidney function should be monitored quarterly. To date, he has tolerated IVIG without any ADRs. However, the nursing staff were advised to decrease his IVIG infusion rate from 300 to 100 mL/h. In addition, he will receive 500 mL of hydration with each infusion (250 mL before and 250 mL during the infusion). To reduce any volume overload, his infusions will also be given every other day rather than on consecutive days. His diabetes will be closely monitored, and he has been referred to a weight loss clinic, as well as a therapist, to manage the onset of depression related to his recent diagnosis and ongoing struggles with weight. If his estimated GFR decreases further and/or he experiences any ADRs related to treatment, he will be reevaluated.

## Discussion

As a result of early reports of renal failure with IVIG, mostly related to sucrose stabilizers, FDA required that all IVIG manufacturers revise their prescribing information to include a black box warning stating that renal dysfunction, acute renal failure, and osmotic nephropathy may occur with IVIG.^43^ All currently approved IVIG products in the US still include this black box warning. In the FDA medical bulletin issued by Epstein et al^43^ in 1999, the authors stated that “reduction in dose, concentration, infusion rate, or all of these in patients at risk of acute renal dysfunction has been proposed in order to reduce the risk.” Like the authors of the current report, they also recommended baseline kidney function measurements before IVIG infusion with repeated measurements at appropriate intervals for those at increased risk of kidney impairment.^43^

The highest rates of systemic adverse events with IVIG, including kidney complications, occur in adult patients with autoimmune, neurological, or hematological disorders receiving high-dose IVIG.^62^ Although most systemic adverse effects present within 10 days of IVIG infusion,^54^ it is also important to consider the potential negative impact to kidney function following long-term therapy. In a 2017 report by Levine and colleagues,^63^ the authors retrospectively analyzed laboratory data from 166 patients who had received 12 months of high-dose IVIG infusions for neurological disorders. In these patients, the GFR measures at 6 and 12 months were compared to baseline values and then averaged to determine the effects on GFR of long-term, high-dose therapy. It is important to note that patients with a history of renal issues were excluded from the study population; the investigators also excluded patients on other drugs that could confound the analysis, including steroids and immunosuppressive medications. In the overall study population, approximately 10.34% of patients had a drop of more than 20% in GFR over 12 months. In addition, for patients receiving Gamunex (Grifols), Privigen (CSL Behring AG), or Bivigam (ADMA Biologics), there was a significant risk of a decrease of more than 20% in GFR. In a study population that was chosen from otherwise healthy individuals, without kidney impairment, diabetes, or potentially nephrotoxic medications, these findings are particularly significant.^63^ The findings emphasize the need to initially evaluate and continue to monitor kidney function in all patients receiving long-term, high-dose IG.

One of the key risk factors for kidney impairment is advanced age (≥65 years) (Figure 1 and eAppendix).^10,64^ The use of IG to treat autoimmune diseases in older individuals is increasing in prevlaence.^65^ In a study by Lozeron and colleagues,^65^ use of IVIG was examined in older individuals who were treated for autoimmune neuromuscular disease. In this study, in patients 60 years of age or older, high-dose, chronic administration of sucrose-free IVIG was associated with ADRs, including kidney failure. The authors noted that higher infusion rates may have had a role and suggested that specific studies may need to be conducted to further explore this correlation. They also recommended that special attention be paid to the maximum daily IVIG dose in older patients.^65^

As detailed herein, there are potential risks related to kidney function during and/or following administration of IG. The goal of this report is to increase awareness of impaired kidney function in patients receiving IG and provide recommendations for screening, monitoring, and management for kidney disease to promote risk prevention and mitigation. Clinicians should evaluate all patients receiving IG for underlying kidney function impairment and CKD; this is of critical importance in patients receiving high-dose IVIG, especially in older patients. If CKD or impaired kidney function is identified, patients receiving IG should be closely monitored and managed to prevent further damage. Identification of the cause of CKD or kidney impairment, if feasible, is strongly encouraged, as IG therapy may need to be modified.

## Conclusions

The majority of adults with CKD or kidney function impairment are unaware of their status. This lack of awareness on the part of patients and their physicians is a major risk factor for worsening of the disease following administration of IG. Therefore, screening of all patients for CKD or kidney function impairment before use of IG, as well as ongoing monitoring and management for patients in whom CKD or kidney function impairment has been identified, is essential. As with all potential ADRs, the best approach is avoidance.

## Supplementary Material

zxac139_suppl_Supplementary_Appendix_S1
