# Supplementary material for: Immune globulin therapy and kidney disease: Overview and screening, monitoring, and management recommendations
Source: Am J Health Syst Pharm. 2022 May 20;79(17):1415–23. doi: 10.1093/ajhp/zxac139 (PMC9389421; doi:10.1093/ajhp/zxac139)
Supplement: zxac139_suppl_Supplementary_Appendix_S1 [file zxac139_suppl_supplementary_appendix_s1.pdf]

Immune Globulin Administration: Screening Algorithm for Potential Kidney Impairment

Identify Patients at Risk for Kidney Impairment

- Pre-existing kidney disease/damage and or family history of kidney disease
- Advanced age (≥ 65 years)
- Diabetes mellitus, hypertension, cardiovascular disease, and/or obesity
- Volume depletion (dehydration or hypervolemia)
- Autoimmune disease (e.g., systemic lupus erythematosus, vasculitis)
- Infections (bacterial, viral, parasitic)
- Paraproteinemia
- Concomitant and/or past nephrotoxic drug usage

Evaluate Kidney Function

- Obtain a renal function panel and estimated glomerular filtration rate (GFR)
- The American Medical Association (AMA)-approved panel includes:
  - Albumin, bicarbonate, calcium (total), chloride, creatinine, glucose, phosphorus, potassium, sodium, and urea nitrogen

Normal Kidney Function

- Estimated GFR is ≥ 60 mL per minute per 1.73 m^2
- Continue to monitor kidney function monthly, quarterly, or annually per degree of concern

Mild-to-Moderate Kidney Dysfunction

- Estimated GFR is 30 to ≥ 59 mL per minute per 1.73 m^2
- Ongoing management and monitoring are required see **Clinical Monitoring and Management Recommendations** (following pages)

Severe Kidney Dysfunction

- Estimated GFR is ≤ 29 mL per minute per 1.73 m^2
- Frequent monitoring and ongoing management are required see **Clinical Monitoring and Management Recommendations** (following pages)

From: Kobayashi RH, Rigas MT. Immune Globulin Therapy and Kidney Disease: Overview and Screening, Monitoring , and Management Recommendations. Am J Health-Syst Pharm. 2022.

Immune Globulin Administration: Clinical Monitoring Recommendations for Patients with Impaired Kidney Function

Table 1. Immune Globulin Administration: Clinical Monitoring Recommendations for Patients With Impaired Kidney Function

Clinical monitoring	Recommendations
Routine laboratory evaluations	• Mild to moderate: renal function panel (Figure 1) and calculation of estimated GFR performed at least quarterly
	• Severe: renal function panel (Figure 1) and calculation of estimated GFR performed monthly or before every IVIG treatment
Changes in weight	• Carefully monitor fluid status, including decreases in urine output, edema, etc.
	• Obese patients should have their dose evaluated and monitored to avoid overestimating.
Medications	• Generally, it is suggested to reassess all concurrently prescribed drugs, particularly in older patients, on a regular basis.
	• Some concomitant medications should be used with caution in patients with impaired kidney function, eg, diuretics, inhibitors of the renin-angiotensin system, and nonsteroidal anti-inflammatory drugs.
Adverse reactions	• Carefully monitor for all adverse drug reactions during and up to 72 hours after infusion; resolve and mitigate them as soon as possible and minimize impact (Box 2).

Abbreviations: GFR, glomerular filtration rate; IVIG, intravenous immune globulin.

From: Kobayashi RH, Rigas MT. Immune Globulin Therapy and Kidney Disease: Overview and Screening, Monitoring , and Management Recommendations. Am J Health-Syst Pharm. 2022.

Immune Globulin Administration: Clinical Management Recommendations for Patients with Impaired Kidney Function

Table 2. Immune Globulin Administration: Clinical Management Recommendations for Patients With Impaired Kidney Function

Clinical management objective	Recommendations
Minimize further kidney injury	• If kidney function estimates show a reduction in creatinine clearance of more than 15%, weight gain of greater than 2.5 pounds, or increased edema, consider the following:<br>◦ Reducing infusion rate—consider a maximum infusion rate of 100 mL/h<br>◦ Spreading out dosing to every other day (or more), as needed (ie, decreasing daily volume)<br>◦ Assessing hydration and oral fluid intake before and after doses<br>◦ Evaluating and adjusting the current regimen of diuretics<br>◦ Carefully monitoring and assessing comorbidities<br>• Delve deeper into the patient's clinical changes and develop a more comprehensive care plan
Minimize the impact of ADRs	• If ADRs occur, adjust the care plan accordingly and consider the following:<br>◦ Introducing the interventions described above to minimize further kidney injury<br>◦ Introducing/modifying an IVIG premedication regimen<br>◦ Changing the concentration of IVIG product or IVIG brand if the ADR is significant<br>◦ Changing the administration location (with additional supervision), if appropriate<br>◦ Switching to SCIG therapy, if the patient is agreeable to this suggestion
Manage psychological and social issues	• Address the psychological impact and social burden of living with chronic kidney disease and consider the following:<br>◦ Therapeutic options for depression and mental health<br>◦ Provision of social services or home health support services<br>◦ Addressing anything else the patient is concerned about, eg, access to support, insurance, supplies, progress towards goals, etc
Minimize the impact of financial issues	• If changes to insurance or the ability to afford out-of-pocket costs occur, consult with the pharmacy intake team to develop a plan to ensure the therapeutic regimen is not interrupted

Abbreviations: ADR, adverse drug reaction; SCIG, subcutaneous immune globulin; IVIG, intravenous immune globulin.

From: Kobayashi RH, Rigas MT. Immune Globulin Therapy and Kidney Disease: Overview and Screening, Monitoring , and Management Recommendations. Am J Health-Syst Pharm. 2022.
